# P-1799. Targeting the Endocannabinoid System: Cannabinoid Receptor 2 Activation Modulates Outcomes in Influenza A Virus Infection – Insights from a Murine Model

**DOI:** 10.1093/ofid/ofaf695.1968

**Published:** 2026-01-11

**Authors:** Ahmed R Alsuwaidi, Junu George, Ifrah Ali, Shreesh Ojha

**Affiliations:** United Arab Emirates University, Al Ain, Abu Dhabi, United Arab Emirates; United Arab Emirates University, Al Ain, Abu Dhabi, United Arab Emirates; United Arab Emirates University, Al Ain, Abu Dhabi, United Arab Emirates; United Arab Emirates University, Al Ain, Abu Dhabi, United Arab Emirates

## Abstract

**Background:**

Emerging evidence suggests that Cannabinoid Receptor 2 (CB2) plays a critical role in regulating inflammation during viral infections. This study investigates the therapeutic potential of CB2 modulation in influenza A virus (IAV) infection using a murine model.

**Methods:**

BALB/c mice were infected intranasally with IAV (A/PR/8/34) and treated with either JWH-133 (CB2 agonist) or AM-630 (CB2 inverse agonist). Four groups were studied: 1) IAV+PBS, 2) IAV+AM-630, 3) IAV+JWH-133, and 4) uninfected PBS controls. AM-630 (2 mg/kg) was administered intraperitoneally (i.p) twice daily starting on day -1 (one day before virus inoculation), while JWH-133 (5 mg/kg) was administered i.p from day 0 through day 5. Mice were monitored daily for survival, clinical scores (ruffled fur, lethargy, labored breathing, and hunched posture), and body weight. CB2 expression in lung tissue was quantified via qRT-PCR.

**Results:**

Mice treated with the CB2 agonist (JWH-133) or inverse agonist (AM-630) survived the 15-day study period, whereas IAV-infected mice without treatment experienced 100% mortality by day 13. Clinical signs appeared later in the CB2 agonist group (day 6) compared to the CB2 inverse agonist (day 4) and IAV-only groups, correlating with delayed weight loss and recovery in the agonist group. CB2 agonist-treated mice regained weight after day 8, similar to the control group, while other infected groups did not recover. Lung CB2 mRNA expression was significantly upregulated in the agonist group but reduced in the antagonist group, confirming receptor activation and blockade.

**Conclusion:**

Pharmacologic activation of CB2 reduces disease severity and enhances survival in influenza-infected mice. These findings highlight CB2 as a promising immunomodulatory target for future antiviral therapies, especially in the context of severe respiratory viral infections like influenza.

**Disclosures:**

All Authors: No reported disclosures

